# A Genome-Wide Association Study Identifies Genomic Regions for Virulence in the Non-Model Organism *Heterobasidion annosum* s.s

**DOI:** 10.1371/journal.pone.0053525

**Published:** 2013-01-16

**Authors:** Kerstin Dalman, Kajsa Himmelstrand, Åke Olson, Mårten Lind, Mikael Brandström-Durling, Jan Stenlid

**Affiliations:** Uppsala BioCenter, Department of Forest Mycology and Plant Pathology, Swedish University of Agricultural Sciences, Uppsala, Sweden; Seoul National University, Republic of Korea

## Abstract

The dense single nucleotide polymorphisms (SNP) panels needed for genome wide association (GWA) studies have hitherto been expensive to establish and use on non-model organisms. To overcome this, we used a next generation sequencing approach to both establish SNPs and to determine genotypes. We conducted a GWA study on a fungal species, analysing the virulence of *Heterobasidion annosum* s.s., a necrotrophic pathogen, on its hosts *Picea abies* and *Pinus sylvestris.* From a set of 33,018 single nucleotide polymorphisms (SNP) in 23 haploid isolates, twelve SNP markers distributed on seven contigs were associated with virulence (*P*<0.0001). Four of the contigs harbour known virulence genes from other fungal pathogens and the remaining three harbour novel candidate genes. Two contigs link closely to virulence regions recognized previously by QTL mapping in the congeneric hybrid *H. irregulare* × *H. occidentale*. Our study demonstrates the efficiency of GWA studies for dissecting important complex traits of small populations of non-model haploid organisms with small genomes.

## Introduction

Genome-wide association (GWA) studies have been used to elucidate the genetic basis for disease traits [1]. The feasibility of GWA studies in plants was demonstrated by Atwell and colleagues [2] who tested 107 phenotypes in *Arabidopsis thaliana* inbred lines, and has also been used to investigate the genetic basis of resistance in different crops or various traits in wild plants and trees [3]. So far, very few GWA studies have been applied to fungi, even though the tools required are available and fungi have the advantages of small genomes, a homokaryotic (haploid) stage, a sexual life cycle and the ability to multiply genotypes for reproducibility. In *Saccharomyces cerevisiae* GWA mapping of mtDNA copy number identified one significant SNP [4] and population based GWA analysis of clinical vs. nonclinical yeast identified several genetic loci associated with the clinical background [5].

However, several different genetic traits have been studied in fungi using quantitative trait loci (QTL) analysis. In *Pleurotus ostreatus*, Santoyo et al. [6] located QTLs linked to lignin-degrading enzymatic activities on six different chromosomes. A QTL associated with pathogenicity on wheat has been identified in the *Fusarium* head blight fungus *Gibberella zeae* [7]. In *Nectria haematococca* MPI, several QTLs for virulence on pumpkin have been found [8]. The genetic components controlling virulence in the necrotrophic forest pathogen *Heterobasidion annosum* (Fr.) Bref. sensu lato (s.l.) have been studied by QTL mapping of fungal growth within sapwood and induced lesion length in phloem in Norway spruce (*Picea abies*) and Scots pine (*Pinus sylvestris*) [9]. Four QTLs, for both lesion length and growth in sapwood on spruce and pine were found located within the same linkage group, while two other pine-specific QTLs associated with growth or lesion length were located to two other groups [9]. In addition to nuclear genetic factors, the virulence has been shown to be influenced by the mitochondrial genome using hybrids of *H. irregulare* × *H. occidentale* [10]. The advantage of using GWA studies over QTL mapping is that no mapping population needs to be generated. Furthermore, because many more recombination events have occurred in natural populations compared to in a single crossing, the linkage blocks are expected to be smaller than in QTL mapping, which implies a higher map resolution [3].

Population genomics and genome-wide mapping have revealed recent hybridization and genetic introgression between the pathogenic fungi *Coccidioides posadasii* and *C. immitis* [11] while a survey of the domesticated and wild yeasts *Saccharomyces cerevisiae* and *S. paradoxus* showed that phenotypic variation correlated broadly with global genome-wide phylogenetic relationships [12]. By SNP identification and whole-transcriptome sequencing, Ellison et al. [13] discovered two recently diverged populations of *Neurospora crassa* in which they identified genomic islands of high divergence that may be the result of local adaptation to a temperature difference between the two populations.

The necrotrophic pathogen *H. annosum* s.l. causes severe damage in coniferous forests throughout the Northern Hemisphere [14]. *H. annosum* s.l. is a complex consisting of five species with different but partially overlapping host preferences [15], [16]. *H. annosum* sensu stricto (s.s.) mainly infects *Pinus* spp. but is also able to attack *Picea* spp. and other soft and hard wood tree species throughout Europe [17]. The economic losses due to devaluation of saw timber because of stem rot, reduced tree growth and tree mortality reach 1 billion Euros yearly for European forest owners. In addition, *H. annosum* s.l. challenges the coniferous forests potential to act as a carbon sink by releasing CO_2_ from decayed wood.

In this study, we identify novel and well-known virulence genes in the conifer pathogen *Heterobasidion annosum* s.s. through GWA studies based on large scale single nucleotide polymorphism (SNP) identification and typing through next generation sequencing. Our results demonstrate the efficiency of this technique in a haploid non-model organism.

## Results

### Generation of a Genomic Data Set

We sequenced the genomes of 23 haploid *H. annosum* s.s. isolates with an Illumina Genome Analyzer. We obtained paired end reads from a 400-base pair (bp) insert library from three of the isolates (90211/2, Sä_16-4 and W_15), while 36-bp single end reads were generated from the remaining 20. The number of reads ranged between 3.2 and 8.9 million for the single end read isolates, and between 12.3 and 15.3 million for the paired end read isolates (Table 1). A combined reference genome was *de novo* assembled from the paired end sequenced isolates using the Velvet assembler [18], [19]. The assembly resulted in a reference genome of 56,195 contigs with an N50 of 41,157 bp and a median coverage of 38.7×. The number of contigs of at least 1000 bp was 2330, which comprise in total 30,569,260 bp (internal gaps excluded). These contigs were used as reference sequences when sequence reads of each isolate were assembled including the three used in the de novo assembly. The assembly of the individual isolates was achieved through mapping towards the combined reference genome using MOSAIK (http://bioinformatics.bc.edu/marthlab/Mosaik). For each isolate, 48% to 75% of the sequence reads were aligned to the reference and the coverage ranged between 80% and 94% with gap regions included (Table 1). The mean read coverage was between 2.6× and 12.6× of the individual isolates. There were 4,398,305 positions where all 23 individuals had sequence coverage of at least 2×. In these positions, a total of 64,055 SNPs were found and 33,018 of these were not singletons. We tested our SNP panel for population structure with the program Structure [20], [21], [22], using one SNP per contig. No signs of population structure were detected.

**Table 1:** Sequence data for the fungal isolates.

Isolate	Number of reads	Aligned (%^a^)	Bp covered in %^b^	Mean coverage (×)
87087/8	8085500	5663815 (70)	91.8	6.5
90231/2	7709202	5812383 (75.4)	91.7	6.7
87068/2	4881680	3350754 (68.6)	87.7	4.0
Rb_28-20	5745116	3869565 (67.4)	88.8	4.6
W_15^c^	15259254	10853302 (71.1)	94	12.5
V5:91_C4	6538484	4670833 (71.4)	91	5.4
91202/2	8785361	6411227 (73)	91.7	7.4
92153/1	4665945	3205708 (68.7)	87	3.8
92182/1	8387492	4033923 (48.1)	86.3	4.7
93030/1	5324851	3675732 (69)	88.3	4.3
Fr_38-9	5736328	3943813 (68.8)	89.1	4.7
AR_18	3984153	2754913 (69.1)	86.4	3.3
Sä_34-1	7428905	5504802 (74.1)	91	6.3
V12:56_C4	4820594	3162468 (65.6)	86.7	3.7
MJT155_b-4	8306930	6038861 (72.7)	91.5	6.9
V3:47_C4	3219690	2181339 (67.7)	80.2	2.6
Rb_30-3	3889667	2780017 (71.5)	86.8	3.3
90211/2^c^	14675726	10950439 (74.6)	93.9	12.6
93014/1	8724653	6316731 (72.4)	91.9	7.3
FW2-2	4454412	3123319 (70.1)	86.4	3.7
Sä_16-4^c^	12338577	9018462 (73.1)	93.9	10.4
L12-1	5661520	3239493 (57.2)	85.4	3.8
V13:53_C2	4385406	3034864 (69.2)	87.8	3.6

a Percent aligned compared with total number of reads.

b Coverage on reference sequence, Sä16-4, 90211-2 and W15 combined.

c Paired-end sequenced isolates.

### Fungal Virulence on Pine and Spruce Differs Significantly between Isolates

Virulence of *H. annosum* s.s. on Scots pine and Norway spruce was measured both as lesion length formed under the bark and fungal growth in the sapwood of the plants. The average infection success was 90% for each plant species and ranged between 30% and 100% for individual isolates (Table 2). There was a significant difference in virulence between isolates on pine and on spruce, measured both as lesion length and fungal growth in sapwood (*P*<0.05, ANOVA). In spruce, fungal growth (SFG) was normally distributed. However, the measurements for growth in pine (PFG) and lesion length in both spruce (SLL) and pine (PLL) had to be transformed to generate a normal distribution. A correlation between lesion length and fungal growth for spruce (*R* ^2^ = 0.6684) was found, but not for pine. A weak but still significant correlation (*R* ^2^ = 0.2363, *P* = 0.0187) was found between PFG and SFG.

**Table 2:** *Heterobasidion annosum s.s*. virulence analysis on *Picea abies* and *Pinus sylvestris*.

Isolate^a^	Pine, lesion^b^		Pine, growth^b^		Spruce, lesion^b^		Spruce, growth^b^	
	Mean (mm)	Successful^c^	Mean (mm)	Successful^c^	Mean (mm)	Successful^c^	Mean (mm)	Successful^c^
87087/8	33.0±37.0	9	2.8±5.7	9	5.9±4.4	9	1.1±2.2	9
90231/2	6.3±5.3	9	1.1±3.3	9	1.0±1.7	3	3.3±2.9	3
87068/6	35.4±61.4	9	7.0±8.6	10	6.1±2.3	9	6.7±10.3	9
Rb_28-20	16.4±31.1	9	9.5±9.0	10	5.3±3.4	10	21.5±9.1	10
W15	9.3±5.5	10	20.5±14.8	10	6.6±3.0	10	23.0±13.0	10
V5:91_C4	10.3±6.1	10	9.0±5.2	10	9.6±6.6	10	26.0±13.7	10
91202/2	11.8±14.3	9	16.1±17.1	9	8.2±5.4	9	27.0±12.3	10
92153/1	16.6±16.4	10	11.5±9.8	10	6.6±1.9	9	27.5±12.3	10
92182/1	17.7±18.1	10	20.5±16.8	10	6.5±4.9	10	28.0±11.8	10
93030/1	9.1±7.5	8	11.0±9.7	10	9.7±7.1	10	29.0±8.4	10
Fr_38_9	19.9±26.6	10	26.5±21.4	10	6.3±4.0	10	30.0±15.3	10
AR_18	14.1±9.2	10	20.0±14.3	10	7.7±3.6	9	34.5±14.0	10
Sä_34-1	18.4±24.4	10	17.5±11.8	10	11.3±8.2	10	35.5±12.8	10
V12:56_C4	6.1±5.1	8	5.0±4.4	9	14.6±12.0	8	35.5±12.6	8
MJT_155b-4	12.5±15.1	10	20.0±11.1	10	9.7±5.5	10	36.0±13.3	10
V3:47_C-4	9.1±6.5	10	19.0±16.8	10	7.3±4.8	10	36.0±9.9	10
Rb_30-3	17.7±19.1	10	11.0±10.8	10	12.8±18.7	10	37.0±11.6	10
90211/2	9.4±6.9	10	14.0±16.0	10	16.0±11.2	9	38.0±16.2	10
93014/1	7.3±4.2	10	21.5±14.2	10	7.6±3.5	10	38.0±11.4	10
FW2_2	18.2±23.0	9	31.1±23.6	9	10.4±6.5	10	39.5±7.3	10
Sä_16-4	24.6±38.1	10	29.0±24.0	10	11.8±6.0	9	39.5±12.8	10
L12-1	6.1±3.9	9	12.5±12.8	10	10.0±3.3	10	40.5±9.0	10
V13:53_C2	9.2±3.9	6	4.2±3.8	6	41.2±24.0	5	62.0±19.6	5

a Isolates are sorted from low to high mean growth in sapwood of spruce.

b Virulence was measured as lesion length and growth in sapwood up- and downstem from the wound combined in mm, ± s.d.

c Number of successful infections out of 10.

### Twelve SNPs are Significantly Associated with Fungal Virulence

**Figure 1:**
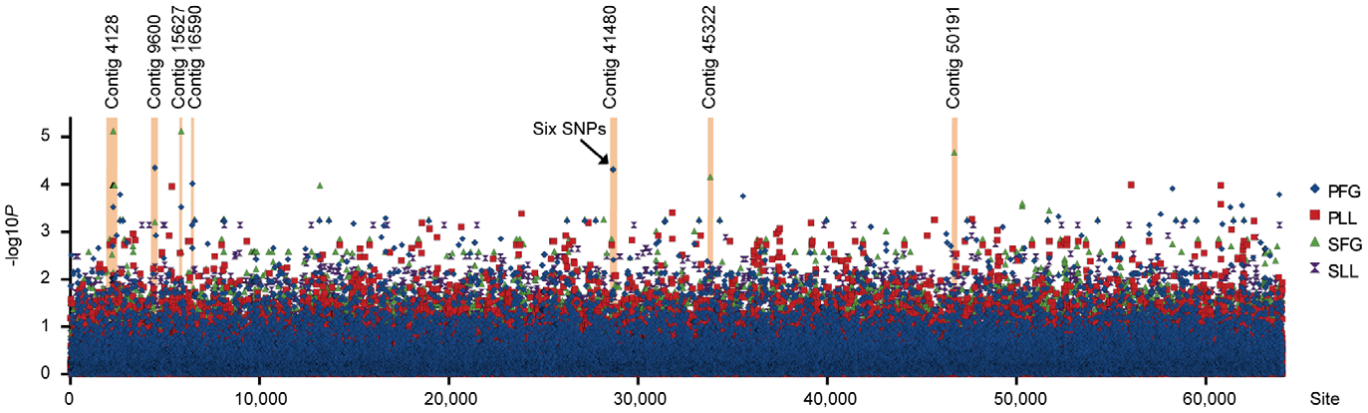
Overview of *p*-values (−log10 scale) for SNP associations to *Heterobasidion* virulence in spruce and pine. The *p*-values are plotted against the site number from the SNP extraction. The contigs with significant SNPs are indicated with orange lines. Abbreviations: PFG, fungal growth in pine sapwood; PLL, lesion length in pine; SFG, fungal growth in spruce sapwood; SLL, lesion length in spruce.

The general linear model revealed an association between 12 SNP markers and virulence with an adjusted *P*-value lower than 0.0001 (10 000 permutations) (Table 3). Out of these twelve, six SNP markers were found on the same contig (contig 41480); the remaining six SNP markers were distributed on six different contigs (Fig. 1). Four SNP markers were associated with SFG and the remaining eight with PFG. No markers were associated with SLL or PLL.

**Table 3:** Significantly associated SNP markers (*P*_adj≤1.00×10^−4^).

SNP Id	Contig	Position in contig	*P*	*P*_adj^a^	Trait^b^	Genotype Minor/major	Homolog in Hetan2 Scaffold:Position	Protein Id^c^	Gene Ontology	Gene	Substitution	AA change (+strand) Minor/Major
2541	4128	41529	7.50×10^−6^	1.00×10^−4^	SFG	T/C	2:90374	–	–	–	Intergenic	None
31888	41480	4151	4.82×10^−5^	1.00×10^−4^	PFG	G/A	13:580629	148882	0016787, Hydrolase activity	Putative calcineurin	Synonymous	None
31890	41480	4348	4.82×10^−5^	1.00×10^−4^	PFG	T/G	13:580826	148882	0016787, Hydrolase activity	Putative calcineurin	Intron	None
31891	41480	4414	4.82×10^−5^	1.00×10^−4^	PFG	G/A	13:580892	148882	0016787, Hydrolase activity	Putative calcineurin	Synonymous	None
31898	41480	8609	4.82×10^−5^	1.00×10^−4^	PFG	T/C	13:575283	–	–	–	Intergenic	None
31912	41480	11461	4.82×10^−5^	1.00×10^−4^	PFG	T/C	13:569318	482843	0005515, Protein binding; 0008270, Zinc ion binding	Unknown	Non-synonymous	Cys (C)/Arg (R)
31915	41480	12127	4.82×10^−5^	1.00×10^−4^	PFG	T/C	13:569985	482843	0005515, Protein binding; 0008270, Zinc ion binding	Unknown	Synonymous	None
7164	16590	17829	9.63×10^−5^	1.00×10^−4^	PFG	A/G	10:1046969	454667	–	Exopolyphosphatase	Non-synonymous	Phe (F)/Ser (S)
4981	9600	30323	4.46×10^−5^	1.00×10^−4^	PFG	A/G	1:562288	99077	–	*SWI5* transcription factor	Synonymous	None
37656	45322	6635	6.93×10^−5^	1.00×10^−4^	SFG	T/C	3:163145	102298	–	Unknown	Intron	None
6505	15627	792	7.50×10^−6^	1.00×10^−4^	SFG	T/C	13:53143	–	–	–	Intergenic	None
51971	50191	17454	2.12×10^−5^	1.00×10^−4^	SFG	C/T	5:438018	–	–	–	Intergenic	None

a *P*-value adjusted after 10,000 permutations.

b SFG, fungal growth in spruce sapwood, upstem and downstem combined; PFG, fungal growth in pine sapwood, upstem and downstem combined.

c ProteinId number for the homologous gene in Hetan2, http://genome.jgi-psf.org/Hetan2/Hetan2.home.html.

**Figure 2:**
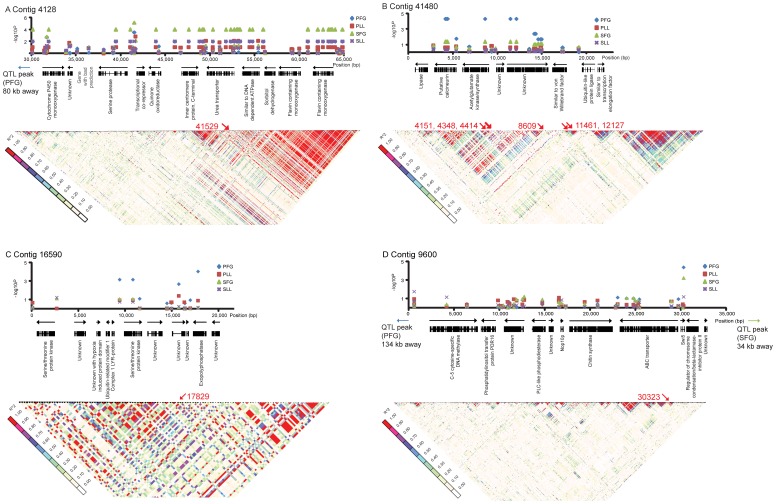
Overview of four genomic regions significantly associated with *Heterobasidion* virulence in spruce and pine. The upper part of each figure plots the *p*-values (−log10 scale) for the four traits (up- and downstem combined) to the genomic position (in bp). Abbreviations: PFG, fungal growth in pine sapwood; PLL, lesion length in pine; SFG, fungal growth in spruce sapwood; SLL, lesion length in spruce. The lower part displays linkage disequilibrium (LD) heat maps. The heat map illustrates the LD value *r* ^2^ from white to red where red indicates high *r* ^2^ -values. Significant SNP markers are in red. (A) Contig 4128; (B) Contig 41480; (C) Contig 16590; (D) Contig 9600.

**Figure 3:**
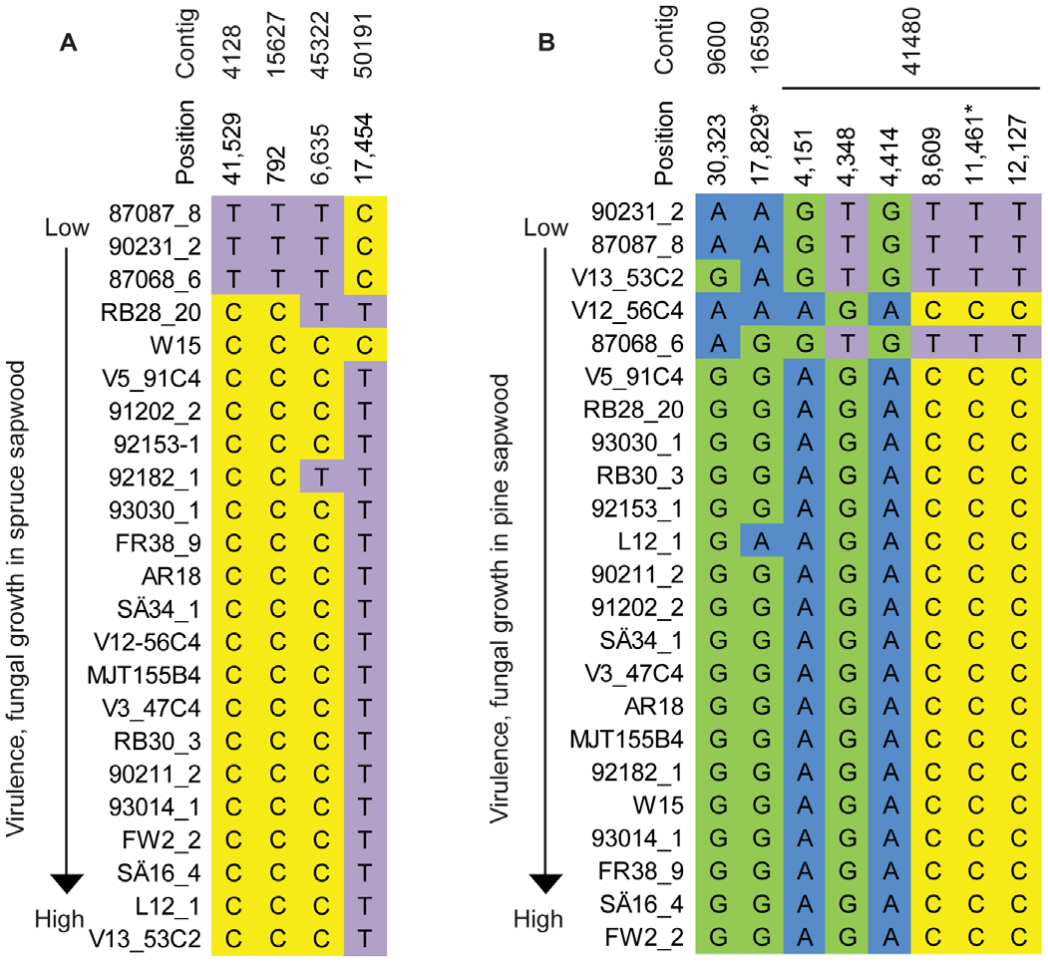
Genotypes for SNPs significantly associated with virulence in spruce and pine. Genotypes for SNPs significantly associated with: (A) fungal growth in spruce sapwood; contig 4128, 15627, 45322, 50191 and (B) fungal growth in pine sapwood; contig 9600, 16590 and 41480. All associations were adjusted by a permutation test, the positions shown have a *p* adj <0.0001. Non-synonymous substitutions are labelled with an asterisk.

The length of the contigs varied between 2.5 kilobase pairs (kb) and 76.5 kb (Table 4) and the SNP density varied in the contigs from 0.4 to 1.7 SNPs per kb (minor allele frequency ≥2/23 individuals; coverage in all individuals). The linkage disequilibrium (LD) heat maps show clear blocks of LD in the two contigs 4128 and 41480 associated with the markers for virulence traits (Fig. 2). In the remaining five contigs the SNP marker associated with virulence was not associated with any LD block (Fig. 2 and Fig. S1). The six SNP markers located in contig 41480 were found in two distinct LD blocks with the same statistical support (*P*<0.0001). The less virulent genotypes were present at low frequencies in the population (Fig. 3).

**Table 4:** Data for the contigs with SNPs associated with virulence.

Contig number	Size of contig (bp)	Number of SNPs Minor allele 2 Coverage 23/23 isolates	Number of SNPs Minor allele 2 Coverage 15/23 isolates
4128	66260	112	764
41480	76532	93	387
16590	46146	19	124
9600	34006	37	518
45322	12543	18	230
15627	2528	3	15
50191	40237	60	510

### Surveying the Genomic Regions Associated with Fungal Virulence

The seven *H. annosum* s. s. contigs containing SNPs associated with virulence were highly homologous to the *H. irregulare* genome (http://genome.jgi-psf.org/Hetan2/Hetan2.home.html) [23] and the gene order within each contig was well conserved. The contigs were not linked among each other and they were distributed over different scaffolds. Contig 4128 had sequence homology to positions 49591–113806 on scaffold 2 in the genome of *H. irregulare*. The LD block harbouring the SNP associated with SFG was located between position 40886 and 66145 in the contig (corresponding to position 89756-113692, scaffold 2 in *H. irregulare*). This region contained nine genes that encode: a serine protease, a transcriptional co-repressor, a quinone oxidoreductase (ToxD), an inner centromere protein, a urea transporter, an enzyme similar to a DNA-dependent ATPase, a sorbitol dehydrogenase and two flavin-containing monooxygenases (Fig. 2A). Marker 2541, associated with SFG, was located in between the two genes; the serine protease and the transcriptional co-repressor (Fig. 2A). Linkage was found between this marker and SNPs in the genes that encode a cytochrome P450, a urea transporter, a DNA-dependent ATPase and a sorbitol dehydrogenase.

Contig 41480 had homology to positions 499810–584466 on scaffold 13 in the genome of *H. irregulare* and they were syntenic, although there was a small break in homology between positions 532643 and 533812, where *H. irregulare* has a transposon inserted. The LD block between 2.2 and 7.0 kb in the contig spanned a putative calcineurin, similar to *S. cerevisiae CNA-1*, and the end of an acetylglutamate kinase/synthase gene (Fig. 2B). Two markers at positions 11461 and 12127 were found in a smaller LD block that was located in a gene of unknown function. An absolute LD (*r* ^2^ = 1) between the significant markers and the rest of the positions in the LD blocks was observed. This linkage is localized to the genes that encode calcineurin, acetylglutamate kinase and the longer unknown gene. Two of the markers associated with virulence in the calcineurin gene were synonymous whereas the third was located in an intron (Table 3). In addition, the small contig 15627 (2.5 kb) also had homology to scaffold 13 (51802–56242) in the genome of *H. irregulare.* This region contains the last 200 bp of a polyprenylsynthetase gene and three unknown genes, out of which two encode secreted proteins.

Contig 16590 had homology to positions 1029321-1073048 on scaffold 10 of the *H. irregulare* genome (Fig. 2C). The significant SNP marker 7164 at position 17829 in the contig was located in a non-synonymous position in an exopolyphosphatase gene (Table 3). It had LD (*r* ^2^ = 1) to one unknown gene at position 14325 and to three intergenic positions.

Contig 9600 had homology to positions 533823-565834 on scaffold 1 in the genome of *H. irregulare*. Only a few, quite small LD blocks were found, the largest being 1.7 kb (Fig. 2D). The significant marker at position 30323 was located to a synonymous position in the transcription factor *SWI5* with an absolute LD (*r* ^2^ = 1) restricted to the same gene at positions 30227, 30247, 30352 and 30602 (Table 3).

Contig 45322 and 50191 had homology to scaffold 3 and 5 of the genome of *H. irregulare*, respectively. The SNP in contig 45322 was localized to a gene of unknown function and the one in contig 50191 was found in an intergenic region (Fig. S1).

## Discussion

Applying GWA analysis to an organism in a haploid stage that can be clonally reproduced in high numbers and phenotyped repeatedly increases the accuracy of the phenotypic measurements and the power of the association analysis with several orders of magnitude as compared to diploids. We demonstrated that as few as 23 haploid individuals could successfully be used to identify convincing associations in small fungal genomes, whereas several hundreds of individuals are needed in diploid organisms with large genomes such as humans and plants [24], [25].

Lind et al. [9] used a mapping population generated from a cross between *H. irregulare* and *H. occidentale* to show that several *H. annosum* s.l. QTLs were associated with fungal virulence on spruce and pine. An improved linkage map [26] located two QTLs on scaffolds 1 and 2 close to or overlapping the *H. annosum* s.s. contigs 9600 and 4128, suggesting that these regions harbour general virulence factors important in several *Heterobasidion* species, e.g. *H. irregulare*, *H. occidentale* and *H. annosum* s.s. Such general virulence regions have previously been suggested by Lind et al. [9] who found one linkage group with QTLs for four virulence traits. The overlapping results between these different studies and mapping methods strengthen the assumption that the regions target important virulence genes. These combined data suggest that virulence in *H. annosum* s. l. is controlled by regions of virulence factors that occur at different positions in the genome, of which some confer general virulence conserved between the different *Heterobasidion* species and other species-specific virulence that have evolved separately. Some of these species-specific genes found in *H. annosum* s.s. may encode virulence factors that are specialized for Scots pine infection. Interestingly, several Norway spruce specific virulence factors were also found with a significant association for SFG, confirming the ability of *H. annosum* s.s. to infect spruce as an alternative host. In addition, this fact may indicate that *H. annosum* s.s. uses different mechanisms to infect spruce than *H. occidentale*.

The success and power of an association study is dependent on the number of SNP markers and on the LD decay. We analysed LD in six contigs using an extended SNP data set and the results indicate a variable LD block size present in our population (300 bp to 31 kb). The very limited LD for the associated SNPs in four of the contigs indicates a high-resolution mapping of these regions. The fact that only part of the genome could be included in the analysis makes it difficult to predict the number of SNPs that are needed for complete coverage in *H. annosum* s.s. Maize has been shown to have an LD decay between 1 and 10 kb that increased with the increase of minor allelic frequencies and with smaller sample sizes [27]. For the diploid maize, small sample size was defined as 25 individuals and a sample size of more than 50 individuals generated no significant differences in the mean *r^2^*. The long LD regions found in *H. annosum* s.s. might therefore partly be an effect of the relatively low number of individuals used.

### Surveying the Genomic Regions Associated with Fungal Virulence

#### Gene models associated with fungal growth in spruce

Markers associated with fungal growth in spruce (SFG) were found to be located in gene models with varying functions (contig 4128, Fig. 2A) as well as to unknown/novel virulence genes (contig 45322, 50191, 15627, Fig. S1). Contig 4128 is located close to a previously known QTL for fungal growth in pine sapwood, which is why the genes found in this region are candidates as conserved, general, virulence factors. The gene encoding a quinone oxidoreductase (contig 4128) is similar to a gene that encodes zinc-binding oxidoreductase ToxD, a host-selective toxin produced by *Pyrenophora tritici-repentis* Pt-1C-BFP [28], with 27% sequence identity and 36% similarity. Ptr ToxD is a zinc enzyme, specific for NADPH that catalyses the one-electron reduction of certain quinones [29]. *Pyrenophora tritici-repentis*, causal agent of tan spot of wheat, is known to produce several additional host-selective toxins, including Ptr ToxA, Ptr ToxB and Ptr ToxC [30].

Serine carboxypeptidases, found in contig 4128, belong to the serine-type catalytic family and use the amino acid as a nucleophile to form an acyl intermediate. They can also act as transferases and are common in viruses, bacteria and eukaryotes [31]. In *Schizosaccharomyces pombe* it was shown that deletion of a serine carboxypeptidase gene, *sxa2*, causes hypersensitivity to the P-factor mating pheromone and a reduction in mating efficiency in M cells [32]. Moreover, in *Cryptococcus neoformans* a deletion mutant of *KIN1*, a serine/threonine protein kinase, was shown to have attenuated virulence [33]. The position of a significant marker between two genes that encode a serine protease and a transcriptional co-repressor indicate that the transcriptional regulation of either of the genes could be affected.

Flavin-containing monooxygenases (FMOs), found in contig 4128, are xenobiotic-metabolizing enzymes that catalyse the oxygenation of nucleophilic nitrogen, sulphur, phosphorous and selenium atoms using NADPH as a cofactor and FAD as a prosthetic group [34]. They have been implicated in the metabolism of several pharmaceuticals, pesticides and other toxicants. *Arabidopsis* mutants overexpressing *FMO1* had an increased basal resistance against *Pseudomonas syringae* pv. tomato and *Hyaloperonospora parasitica*, which suggests that the FMO was involved in detoxifying the virulence factors produced by the pathogens [35]. Another *FMO* was previously found within a QTL for lesion lengths in pine bark close to contig 9600 suggesting a general and conserved role in pathogenicity [9] (Lind M, Dalman K, Olson Å, Brandström-Durling M, Stenlid J in prep.).

#### Gene models associated with fungal growth in pine

Three contigs were found to harbour markers associated with fungal growth in pine (PFG) (contig 41480, 16590, 9600). The general picture of LD for the significant markers in contig 41480 is that it is limited to three genes, calcineurin, acetylglutamate kinase and a longer unknown gene. Therefore these genes, along with exopolyphosphatase (contig 16590) and *SWI5* (contig 9600), are the strongest candidates for the PFG virulence trait. The putative gene for calcineurin found in this study represents a strong candidate for virulence signalling in *H. annosum* s.s. Calcineurin is a phosphatase regulated by Ca^2+^ and calmodulin, and is heavily involved in the calcium-dependent signal transduction pathways of many processes in eukaryotes, such as T cell activation, muscle hypertrophy, memory development, glucan synthesis, ion homeostasis and cell cycle control [36]. Furthermore, the protein confers a conserved function for virulence in several fungi; e.g. *Candida albicans* [37], *Ustilago maydis* [38] and *Sclerotinia sclerotiorum* [39]. The plant pathogen *Botrytis cinerea* calcineurin-responsive transcription factor CRZ1 was found to be required for penetration of plant surfaces [40]. In the rice blast fungi *Magnaporthe oryzae*, deletion mutants for the genes encoding a calcineurin-responsive transcription factor had a reduced virulence due to a defect in host penetration [41], [42].

A gene encoding *N*-acetylglutamate was found in contig 41480. *N*-acetylglutamate is involved in the biosynthesis of arginine in prokaryotes, lower eukaryotes and plants [43]. An insertional mutation or deletion of genes encoding *N*-acetylglutamate lead to reduced virulence in *Gibberella zeae* (anamorph, *Fusarium graminearum*) [43] and in *Colletotrichum higginsianum* [44].

Exopolyphosphatases (PPXs), found in contig 16590, hydrolyse and release the terminal phosphate from linear polyphosphate containing three or more phosphoanhydride bonds [45]. Together with polyphosphate kinases (PPKs), responsible for polyphosphate synthesis, PPXs maintain the dynamic balance of the polyphosphate level. Polyphosphate is essential for growth of cells, responses to stresses and virulence in several bacteria [45]. Mutants lacking PPX in the human pathogen *Neisseria meningitidis* had an increased resistance to complement-mediated killing [46].

In contig 9600 the significant marker was located to a *SWI5* transcription factor. The homolog in *C. albicans*, *CaACE2*, was shown to affect virulence and deletion mutants of *CaACE2* were avirulent in a mouse model [47]. Strong QTLs for lesions in pine and spruce bark, and growth in spruce sapwood, are overlapping this contig [9](Lind M, Dalman K, Olson Å, Brandström-Durling M, Stenlid J in prep.). The corresponding peaks of these QTLs were at approximately 400 kb (pine) and 600 kb (spruce) respectively on scaffold 1 in the genome of *H. irregulare*. Candidate genes from this region are likely to play an important part in general *Heterobasidion* pathogenicity.

### Conclusions

In this study we show that GWA studies are useful for dissecting important complex traits of non-model organisms, in particular those with small genomes and a haploid life style. We characterized seven genomic regions associated with fungal growth in the sapwood of spruce and pine and present eight candidate virulence genes that encode quinone oxidoreductase (ToxD), serine carboxypeptidase, two flavin-containing monooxygenases, calcineurin, acetylglutamate kinase, exopolyphosphatase and the *SWI5* transcription factor. Of these, all except calcineurin, acetylglutamate kinase and exopolyphosphatase were found very close to or directly overlapping with previously known virulence QTLs.

## Methods

### Plant and Fungal Material

Two-year-old Norway spruce (*Picea abies*) and Scots pine (*Pinus sylvestris*) plants, originating from Latvia and Gotthardsberg in Sweden, respectively, were washed and planted in 2 L pots with fertilized peat. The plants were grown for one month in the greenhouse at 20°C before inoculation. The 23 haploid *H. annosum* s.s. isolates used in this study originated from field studies from different geographic locations in Europe (Table 5). They were all single spore isolates isolated from basidiospores or conidiospores. All isolates were grown on Hagem medium [48] at 21°C in darkness for one week whereafter autoclaved pine wood blocks (5×5×5 mm) were placed on the mycelia. The cultures were incubated for a further four weeks to allow thorough colonization of the wood blocks.

**Table 5:** Haploid isolates of *Heterobasidion annosum* s.s. used in the study.

Isolate^a^	Geographic origin	Host	Coll.^b^
87087/8	Wettingen, Zürich, Switzerland	*Pinus* sp.	OH
90231/2	Stolptsky, Belarus	*Juniperus* sp.	KK
87068/6	Pistoia, Italy	*Pinus nigra*	PC
Rb_28-20	Ramsåsa, Sweden	*Picea abies*	JS
W_15	England, UK	Unknown	JS
V5:91_C4	Vedby, Sweden	*Larix eurolepis*	JS
91202/2	Tartu, Estonia	*Pinus* sp.	KK
92153/1	Bayern, Happerg, Germany	*Picea abies*	OH
92182/1	Stors, Hillerød, Denmark	*Picea abies*	IT
93030/1	Bayern, Happerg, Germany	*Picea abies*	OH
Fr_38-9	Frossarbo, Sweden	*Picea abies*	JS
AR_18	England, UK	*Pinus sylvestris*	JS
Sä_34-1	Sätuna, Sweden	*Picea abies*	JS
V12:56_C4	Vedby, Sweden	*Larix eurolepis*	JS
MJT_155b-4	Mjölby, Sweden	*Pinus sylvestris*	JS
V3:47_C-4	Vedby, Sweden	*Larix eurolepis*	JS
Rb_30-3	Ramsåsa, Sweden	*Picea abies*	JS
90211/2	Negoreloje, Belarus	*Pinus sylvestris*	KK
93014/1	Bayern, Happerg, Germany	*Picea abies*	OH
FW2-2	Scotland, UK	*Picea sitchensis*	JS
Sä_16-4	Sätuna, Sweden	*Picea abies*	JS
L12-1	Scotland, UK	*Picea sitchensis*	JS
V13:53_C2	Vedby, Sweden	*Larix eurolepis*	JS

a Single spore isolates from basidiospores or conidiospores.

b Collectors: JS – J. Stenlid; KK – K. Korhonen; OH – O. Holdenrieder; PC – P. Capretti; IT – I. Thomsen.

### Virulence Assay

The spruce and pine plants were inoculated with an *H. annosum* s.s. isolate by cutting a small window (5×10 mm) in the cambium of the plant, halfway between two nodes, inserting a colonised wood block in the wound and then wrapping it with Parafilm®. The experiment was executed in blocks: two spruce and two pine plants were inoculated with each of the 23 fungal isolates each day for five consecutive days, so that in total ten spruce and ten pine plants were inoculated with each isolate. As a control, ten spruce and ten pine plants were inoculated with sterile wood blocks. After four weeks the plants were harvested and the lesion lengths upstem and downstem from the wound was measured. The stems were then cut into 5-mm pieces and placed on wet filter paper in Petri dishes for 7 days. Growth within sapwood upstem and downstem from the wound was scored by the presence of conidiophores of *H. annosum* s.s. Inoculation was considered to have been unsuccessful in plants that showed no visible lesion, no fungal growth in sapwood and no conidia in the wound: these stems were discarded.

### Statistical Tests for the Virulence Assay

A one-way ANOVA, performed using Minitab® 15.1.0.0, was used to test the difference in virulence between isolates. The measured values for virulence were normally distributed for SFG, but not for PFG or for SLL and PLL. To obtain a normal distribution for the three latter traits, 2 were added to every measure whereupon they were log transformed. The resulting mean values for all four traits were used in the regression analysis and in the association mapping.

### SNP Extraction

*Heterobasidion annosum* s.s. mycelia were grown and DNA extracted according to Lind et al. [49]. All library preparations and sequencing were performed at the SNP&SEQ Technology Platform of Uppsala University Hospital on the Illumina Genome Analyzer according to Illumina’s standard protocol. For three of the isolates (90211/2, Sä_16-4 and W_15), libraries with an insert length of 400 bp were sequenced from both ends (paired end).

Reads from the three paired-end-sequenced isolates were *de novo* assembled using Velvet version 0.7.60 (hash length 21 bp) [18], [19]. Contigs larger than 1000 bp were used as reference sequences. Reads from each isolate (including those isolates that were used for the de novo assembly) were aligned to the reference contigs using MOSAIK version 1.0.1384 (http://bioinformatics.bc.edu/marthlab/Mosaik). Two mismatches per read were allowed using a hash size of 10; only the uniquely aligned reads were accepted. The SAMtools program (pileup -c) [50], [51] and an in-house Python script were used to find SNPs. Each SNP had to have a SNP quality of at least 10 according to Li et al. [51]. Only sites where all individuals had coverage of two reads or more were used. The data was submitted to the Sequence Read Archive, SRA (SRA050098).

### Population Structure Analysis

Structure within the sample population was explored using the program STRUCTURE [20], [21], [22]. To minimise the impact of linkage between SNPs in the structure analysis, we selected a single SNP representing each contig. These were analysed with different assumed population subdivisions in the range from one to four subpopulations in a model assuming the SNPs were not linked and the organism haploid.

### Association Mapping and Linkage Disequilibrium Analysis

The association between SNP markers and virulence estimates was analysed using TASSEL version 2.1 [52]. A general linear model (GLM) approach assuming a completely random mating population was used. The imported phenotypic data consisted of transformed and non-transformed mean values of the four traits. The association analysis was performed in two rounds; first using the SNP dataset from the whole genome and then using an extended dataset from each selected genomic region found to be significantly associated with virulence in the first round. The SNP dataset for the first round consisted of SNPs with a minor allelic frequency of 9% and a minimum coverage of 100% and in the second round a minor allelic frequency of 9% and a minimum coverage of 65%. A permutation test with 10 000 replicates, implemented in TASSEL, was used to correct the *p*-values for each site. Linkage disequilibrium (LD) heat maps, based on *r^2^* calculations implemented in TASSEL, were constructed for the selected genomic areas using the second SNP dataset.

### Alignment and Annotation

The SAMtools pileup -c output [50] and an in-house Python script were used to retrieve the consensus sequences from the selected genomic regions of all isolates. Corresponding genomic regions were identified using BLAST searches of the complete sequenced genome of *H. irregulare*, isolate TC32-1 (http://genome.jgi-psf.org/Hetan2/Hetan2.home.html). The gene annotations found in TC32-1 were transferred to the reference sequence using PROT_MAP, FGENESH-2 (SoftBerry, Mount Kisco, NY) and Artemis [53]. The alignments were further analysed for synonymous and non-synonymous substitutions using MEGA version 4 [54].

## Supporting Information

Figure S1 **Overview of two genomic regions significantly associated with** ***Heterobasidion*** **virulence in spruce and pine.** The upper part of each figure plots the *p*-values (−log10 scale) for the four traits (up- and downstem combined) to the genomic position (in bp). Abbreviations: PFG, fungal growth in pine sapwood; PLL, lesion length in pine; SFG, fungal growth in spruce sapwood; SLL, lesion length in spruce. The lower part displays linkage disequilibrium (LD) heat maps. The heat map illustrates the LD value *r* ^2^ from white to red where red indicates high *r* ^2^ -values. Significant SNP markers are in red. (A) Contig 45322; (B) Contig 50191.
(TIF)
