# Supplementary figures and images for: A Genome-Wide Association Study Identifies Genomic Regions for Virulence in the Non-Model Organism Heterobasidion annosum s.s
Source: PLoS One. 2013 Jan 16;8(1):e53525. doi: 10.1371/journal.pone.0053525 (PMC3547014; doi:10.1371/journal.pone.0053525)

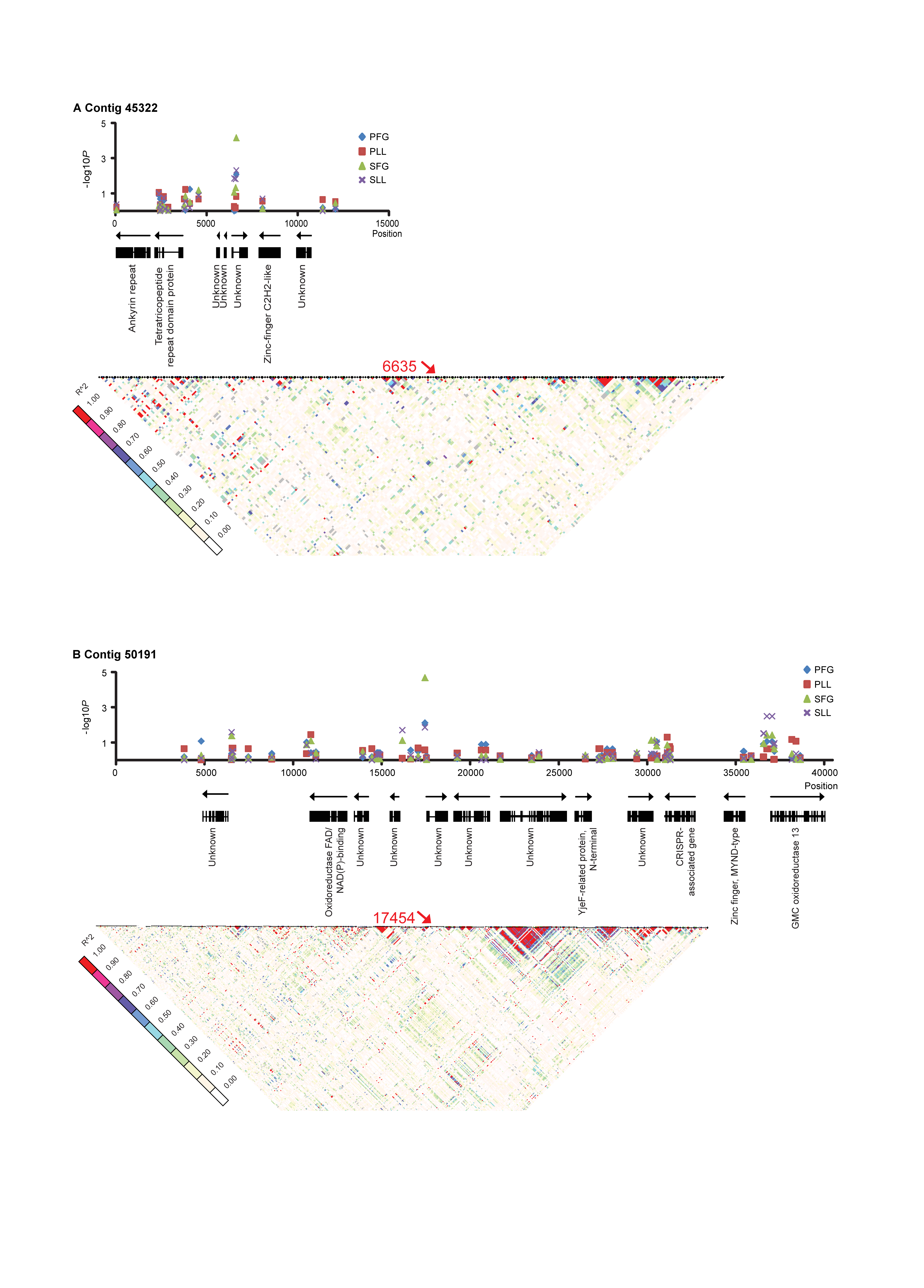

Supplement: Figure S1 — Overview of two genomic regions significantly associated with Heterobasidion virulence in spruce and pine. The upper part of each figure plots the p-values (−log10 scale) for the four traits (up- and downstem combined) to the genomic position (in bp). Abbreviations: PFG, fungal growth in pine sapwood; PLL, lesion length in pine; SFG, fungal growth in spruce sapwood; SLL, lesion length in spruce. The lower part displays linkage disequilibrium (LD) heat maps. The heat map illustrates the LD value r 2 from white to red where red indicates high r 2 -values. Significant SNP markers are in red. (A) Contig 45322; (B) Contig 50191. (TIF) [file pone.0053525.s001.tif]
